# Facing Your Fear in Immersive Virtual Reality: Avoidance Behavior in Specific Phobia

**DOI:** 10.3389/fnbeh.2022.827673

**Published:** 2022-04-27

**Authors:** Florian P. Binder, Dorothee Pöhlchen, Peter Zwanzger, Victor I. Spoormaker

**Affiliations:** ^1^Department of Translational Research in Psychiatry, Max Planck Institute of Psychiatry, Munich, Germany; ^2^International Max Planck Research School for Translational Psychiatry (IMPRS-TP), Max Planck Institute of Psychiatry, Munich, Germany; ^3^kbo-Inn-Salzach-Hospital, Clinical Center for Psychiatry, Psychotherapy, Psychosomatic Medicine and Geriatrics, Wasserburg am Inn, Germany; ^4^Department of Psychiatry and Psychotherapy, Ludwig Maximilian University of Munich, Munich, Germany

**Keywords:** anxiety disorders, specific phobia, avoidance behavior, immersive, virtual reality, arachnophobia, spider phobia, exposure

## Abstract

Specific phobias are the most common anxiety disorder and are characterized by avoidance behavior. Avoidance behavior impacts daily function and is proposed to impair extinction learning. However, despite its prevalence, its objective assessment remains a challenge. To this end, we developed a fully automated experimental procedure using immersive virtual reality. The procedure contained a behavioral search, forced-choice, and an approach task with varying degrees of freedom and task relevance of the stimuli. In this study, we examined the sensitivity and feasibility of these tasks to assess avoidance behavior in patients with specific phobia. We adapted the tasks by replacing the originally conditioned stimuli with a spider and a neutral animal and investigated 31 female participants composed of 15 spider-phobic and 16 non-phobic participants. As the non-phobics were quite heterogeneous in terms of their Fear of Spiders Questionnaire (FSQ) scores, we subdivided them into six “fearfuls” that had elevated FSQ scores, and 10 “non-fearfuls” that had no fear of spiders. The phobics successfully managed to complete the procedure and showed consistent avoidance behavior across all behavioral tasks. Compared to the non-fearfuls, which did not show any avoidance behavior at all, the phobics looked at the spider much more often and clearly directed their body toward it in the search task. In the approach task, they hesitated most when they were close to the spider, and their difficulty to touch the spider was reflected in a strong increase in right hand acceleration changes. The fearfuls showed avoidance behavior depending on the tasks: strongest in the search task and weakest in the approach task. Additionally, we identified subjective valence ratings of the spider as the main influence on both objective avoidance behavior and subjective well-being after exposure, mediating the effect of the FSQ. In summary, the behavioral tasks are well suited to assess avoidance behavior in phobic participants and provide detailed insights into the process of avoidance.

## Introduction

Anxiety disorders are the most common mental disorders with 14% of the population being affected by an anxiety disorder (Wittchen et al., 2011). Pathological fear or anxiety differs from normal feelings of arousal or nervousness in that the levels of fear and anxiety are out of proportion to the actual danger (American Psychiatric Association, 2013). With a prevalence of 6.4%, specific phobia is the most common anxiety disorder (Wittchen et al., 2011).

Avoidance behavior is a key symptom of anxiety disorders. Individuals with specific phobia intentionally behave in ways that prevent or minimize contact with the phobic object and avoid situations in which the phobic object might appear (American Psychiatric Association, 2013). This avoidance behavior leads to significant distress and impairment in important areas of functioning, such as in their social or occupational environments. In addition, avoidance behavior prevents fear extinction, thus maintaining the fear (Lovibond et al., 2009). Despite its importance, the clinical and scientific investigation of avoidance behavior remains a challenge. In clinical contexts, avoidance behavior is usually assessed by self-monitoring and self-report (Antony et al., 2002; Grös and Antony, 2006), such as with the Acceptance and Action Questionnaire (Hayes et al., 2004; Bond et al., 2011). In anxiety research, avoidance behavior is typically operationalized as a decision captured by pressing buttons (Pittig et al., 2014; Sheynin et al., 2014; Krypotos et al., 2018; Luo et al., 2018) or moving a joystick (Grillon et al., 2006). Another way to assess avoidance behavior in specific phobia, used in both contexts, is the Behavioral Avoidance Test. In this task, subjects are confronted by the feared object and asked to approach it as close as possible (Antony et al., 2002; Grös and Antony, 2006). Although the test yields valid and reliable quantification of avoidance behavior, it is very disturbing for the patients and the provision and implementation is costly, as the feared objects, often animals, must be present and cared for. Therefore, an interesting alternative to the presentation of real feared objects is the presentation of virtual objects in virtual reality (VR).

In early VR-based approaches, artificial scenarios were presented to participants on a screen in front of them, and they navigated them with a joystick or by pressing buttons. In immersive VR (iVR), participants wear VR goggles with a screen for each eye that provides a stereoscopic first-person view of the artificial environment. The position and rotation of the goggles are tracked and reflected in the artificial environment. Thus, participants get a three-dimensional all-round view and navigate using head movements as they would in their natural environment. The main advantages of iVR are the full controllability of the environment, the very high standardization, the ease of switching between different environments and the possibility to record all relevant data for later analysis. To use these advantages in the Behavioral Avoidance Test, Mühlberger et al. (2008) developed a Virtual Reality Behavioral Avoidance Test for arachnophobia. In this test, female spider-phobic participants sat on a chair wearing VR goggles and used a joystick to move as close as possible to a spider in the virtual room. They showed that higher levels of fear of spiders were associated with less approach behavior (Mühlberger et al., 2008).

Immersive virtual reality was used to investigate avoidance behavior in spider-fearful participants by Rinck et al. (2010). Their participants wore VR goggles and navigated by walking around freely. The task was to search for certain paintings within a virtual museum with several rooms, some of which contained spiders. They showed that spider-fearful participants had an increase in state anxiety, spent more time looking at spiders, and were more engaged in spontaneous avoidance behavior toward spiders. In our previous work (Binder and Spoormaker, 2020), we used the iVR approach to examine if we could objectively quantify avoidance behavior after Pavlovian fear conditioning. In our setup, the healthy participants wore VR goggles and a full-body motion tracking system that provided a virtual representation of participant’s body in the artificial environment. Participants used their naturally controlled body representation to interact in VR. We developed a behavioral search, a forced-choice, and an approach task to cover a broader range of human behavior and to examine the consistency of avoidance behavior. The tasks differ in degrees of freedom, gamification level, and task relevance of the conditioned stimuli. Higher degrees of freedom lead to more ecological validity, but also increase complexity and analytical flexibility. Gamification was used to get participants to move while distracting them and allowing the capture of more implicit behavior. The task relevance of the conditioned stimuli is usually high in fear conditioning paradigms, but low task relevance can be beneficial in detecting relationships between task performance and trait anxiety (Dodd et al., 2017) or phobic fear (Okon-Singer et al., 2011). We observed that in healthy controls, the behavioral search task with low task relevance of the conditioned stimuli, the highest degrees of freedom, and distraction by gamification elements, was the most sensitive to detect avoidance behavior for the conditioned stimuli. The forced-choice task showed a bimodal distribution with some participants consistently avoiding the conditioned stimuli, and others displaying no initial avoidance behavior. However, the approach task was only sensitive to “strong” avoidance behavior after additional reinforcement.

The goal of this study was to examine the sensitivity and feasibility of these tasks to assess avoidance behavior in patients with specific phobia. This would allow a standardized quantification of avoidance behavior in patients and the online assessment of avoidance behavior during potentially automated therapeutic sessions. Furthermore, we aimed to characterize avoidance behavior in more detail, as our setup allows the continuous tracking of head, limb and body movements in VR and heart rate, as well as pupil size and gaze behavior by incorporation of an integrated eye-tracking system. Heart rate is regulated by the autonomic nervous system and has been proposed to reflect physiological arousal (Berntson et al., 2016), one of the dimensions on which emotions are commonly described (Lang, 1985). Pupil size has been associated with a range of cognitive and affective processes, from cognitive effort to uncertainty and memory (Mathôt, 2018). In threat-related contexts, pupil dilation appears to reflect the salience of stimuli and increases with increasing arousal of stimuli in a valence-independent manner (Hess and Polt, 1960; Aston-Jones and Cohen, 2005; Bradley et al., 2008). Gaze behavior reflects attentional processes that have been shown to be altered in patients with spider phobia (Abado et al., 2020). We focused on spider phobia in our experiments, as the phobic object is well defined, and the prevalence is high. Around one third of the population has a strong dislike of spiders (Davey, 1991; Muris et al., 1997) with females being more affected than males, at rates of 5:1 (Fredrikson et al., 1996). To contrast with the spider, we used a turtle as a neutral control stimulus to detect response differences to these stimuli. With this setup, we could evaluate which tasks and variables reveal the most robust differences between phobics and matched healthy controls, if there were variables that would provide a “clean break” between affected and non-affected individuals, while simultaneously assessing to what extent participants’ behavior depended on their fear levels.

## Materials and Methods

### Participants

In this study, we investigated 32 female participants. One participant was excluded from the analyses because she had severe fear of the control stimulus, resulting in 31 participants (age: *M* = 24.5, *SD* = 4.3, range = 18–35).

Participants were recruited between December 2020 and August 2021 through announcements for people with fear of spiders and announcements for healthy controls on our website and social media. Independent of the announcement, all participants filled out an online screening questionnaire to check the inclusion criteria: aged 18–35, healthy, non-smoker, right-handed, non-pregnant, and a Composite International Diagnostic Screener (CID-S; Wittchen et al., 1999) score below five. Additionally, their severity of spider phobia was assessed by the four items Fear of Spiders Screening (Rinck et al., 2002) in order to control severity distribution and to allow stratification into the extremes “very strong fear of spiders” and “no fear of spiders at all.” Eligible participants were automatically redirected for appointment.

**FIGURE 2:**
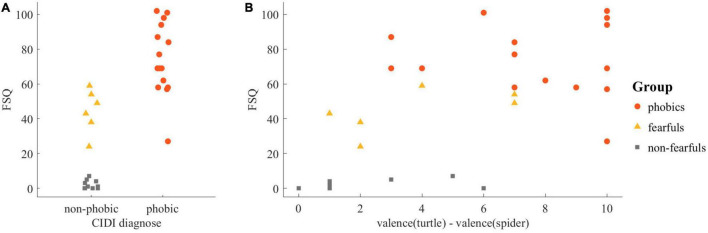
Sample description: **(A)** Diagnosis for spider phobia of the Composite International Diagnostic Interview in association with the Fear of Spiders Questionnaire (FSQ) yielded three clusters that formed the basis for the groups. **(B)** The difference in the valence rating of the turtle and the valence rating of the spider in association with the FSQ.

The participants were assigned to the phobic group or non-phobic group using the Composite International Diagnostic Interview (CIDI; Wittchen and Pfister, 1997). However, as shown in Figure 2A, some of the non-phobic participants had Fear of Spider Questionnaire (FSQ, Szymanski and O’Donohue, 1995) scores above eight, meaning that not all of them were free of fear of spiders (Rinck and Becker, 2007). To be able to examine these differences as well, we further subdivided this non-phobic group and partitioned the whole sample into three groups based on the results of the CIDI and the FSQ score: *phobics* (*N* = 15, age: *M* = 24.9, *SD* = 5.0) who fulfilled the DSM-IV criteria for animal type specific phobia of spiders according to the CIDI; *fearfuls* (*N* = 6, age: *M* = 24.0, *SD* = 5.4) who did not fulfill the DSM-IV criteria but had an FSQ score greater than eight; *non-fearfuls* (*N* = 10, age: *M* = 24.3, *SD* = 2.5) who were both not spider phobic and who had FSQ scores less or equal to eight, as in Rinck and Becker (2007). According to the CIDI, as well as the BDI and CID-S scores, no participant of the non-fearfuls had a psychiatric disorder, and five participants of the phobics had one or two comorbid anxiety disorders (see 1 Comorbidities in Supplementary Material for more details).

The study was conducted in accordance with the Declaration of Helsinki (seventh revision, 2013) and approved by the Local Ethics Committee at the Faculty of Medicine at Ludwig Maximilian University of Munich (reference number: 18–403).

### Procedure

One day before participation, participants filled out an online questionnaire at home consisting of the Big Five Inventory (BFI, Rammstedt and Danner, 2017), Anxiety Sensitivity Index 3 (ASI, Kemper et al., 2009), Trait Anxiety (TAI, Spielberger, 1983), Beck-Depression Inventory II (BDI, Kühner et al., 2007), Fear of Spiders Screening (SAS, Rinck et al., 2002), Fear of Spider Questionnaire (FSQ, Szymanski and O’Donohue, 1995; Rinck et al., 2002), assessment of disgust sensitivity (FEE, Schienle et al., 2002), Short Scale for the Assessment of Locus of Control (IE-4, Kovaleva et al., 2014), Rosenberg’s global Self-Esteem (RSES, Ferring and Filipp, 1996), competence and locus of control (FKK, Krampen, 1991), Sensation Seeking Scales, Form V (SSSV, Beauducel et al., 2003), and the CID-S (Wittchen et al., 1999). Further details on the questionnaires used can be found in Supplementary Table 3.

Due to the Covid19 pandemic, hygiene requirements changed in the meantime and led to some, but not all, participants taking a Covid19 test when they arrived and wearing a FFP2 mask during the preparations and the Composite International Diagnostic Interview (CIDI; Wittchen and Pfister, 1997). No masks were worn during the VR session.

Participants arrived either at 1 pm or at 3:30 pm. They were informed about the procedure and gave their written informed consent. Next, the electrodes for the one channel eMotion Faros 180 electrocardiography device from BioSign and the 18 sensors of the Perception Neuron V2 motion tracking system were attached to the torso, limbs, and head and then calibrated (see Binder and Spoormaker, 2020 for details). The participants put on the HTC Vive Pro + Eye VR goggles and the HTC in-ear headphones and the automated procedure was started. The original headphones of the HTC Vive Pro were not used as they disturbed the functioning of the motion tracking system.

**FIGURE 1:**
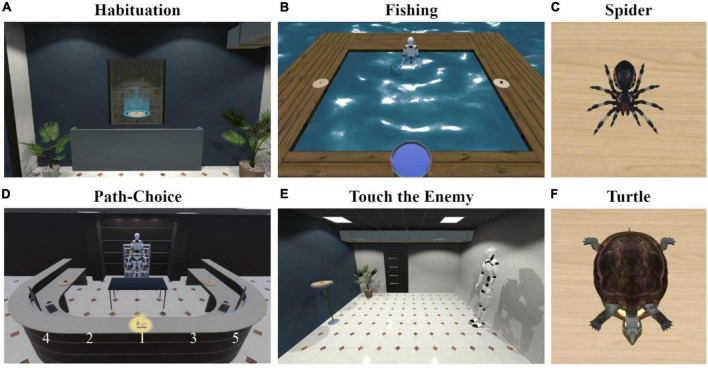
Screenshots of the virtual reality scenes: **(A)** Habituation—first person view of the stimulus habituation with the blue arrow being present, **(B)** Fishing—the behavioral search task, **(C)** Spider—the aversive stimulus, **(D)** Path-Choice—the behavioral forced-choice task, the white numbers indicate the book position of the respective trial, **(E)** Touch the Enemy—the behavioral approach task, **(F)** Turtle—the control stimulus.

From this moment on, all tasks and instructions in iVR were fully automatized, and the participants were instructed not to ask questions, except for urgent ones (no one did). The procedure started with the HTC Vive eye tracking calibration, which set up the inter pupil distance and calibrated the eye-tracking through a two-dimensional five-point calibration. It was followed by a short three-dimensional eye-tracking validation task. Next, pupil size was calibrated by alternately displaying different colors on the screen in the goggles. Afterward, the participants were instructed to behave in the VR as in the real world and not to walk through virtual objects, as they could represent real ones. The introduction continued with some tasks to familiarize participants with navigation and item collection as described in Binder and Spoormaker (2020). At the end of the introduction, the spider and the turtle were sequentially presented in a small side room behind a glass pane (see Figure 1A). It was also demonstrated and explained that the animals were always marked with a big blue arrow above them, pointing down at them. The arrow disappeared once the participant looked at the stimulus. This was done to assure participants that there were no hidden spiders that could surprise them. After each presentation, the participants were asked to rate the valence of the animal on a five-point Likert scale. The introduction was finished after the valence-ratings of the animals. The behavioral tasks as described below (2.4 Behavioral Tasks) followed, in the fixed order: Fishing, Path-Choice, and Touch the Enemy. This order was chosen to gradually decrease the degrees of freedom and increase the task intensity. After the last behavioral task, a final scene followed, in which participants were seated and took off the goggles.

After the VR session all sensors were detached, and the participants filled out the post-VR questionnaire on a tablet device. The questionnaire consisted of a visual anxiety scale (ordinal: 0 = “not at all,” 10 = “extremely anxious”), valence ratings of the animals (ordinal: 0 = “unpleasant,” 10 = “pleasant”), an evaluation of the duration in VR, the Simulator Sickness Questionnaire (SSQ, Kennedy et al., 1993), the Presence Questionnaire 3 (PQ3, Witmer et al., 2005), the iGroup Presence Questionnaire (IPQ, Schubert et al., 1999), and a final question to assess the nervousness on arrival. Finally, the anxiety section of the CIDI was conducted by a trained person (FB). The procedure was completed by filling out the reimbursement form for 30 EUR for participation.

### Virtual Reality

The VR was generated in Unity 3D Pro (version: 2020.2.2f1). We used the same setup and scenarios with a field of 4.6 m × 4.3 m, as in Binder and Spoormaker (2020). Instead of fear conditioned balloons, we used a spider (“Giant Spiders Animated,” version 1.0.0, “spider_hi_004” scaled by 0.03, length: 8 cm, Figure 1C) as aversive stimulus and a turtle (Chinese box turtle, version 1.0.4, “PondTurtleMiddlePoly” scaled by 0.7, size: length: 14 cm, Figure 1F) as neutral stimulus, which were purchased in the Unity 3D asset store.

### Behavioral Tasks

#### Behavioral Search Task (“Fishing”)

The participants were standing in 80 cm deep non-transparent water, surrounded by wooden planks that indicated the borders of the field (see Figure 1B and Supplementary Video 1). They started at the edge, centered in front of one of the long sides, facing the center of the field. They were instructed that there were fish in the water, which could neither be seen directly nor were there any hints indicating their position. The task was to catch them with the hand net in the right hand and to put them into the bucket on the plank opposite the starting position. On each of the left or right wood planks was either the spider or the turtle placed in the center. These positions were counterbalanced between participants. After the instruction, the participants had to wait 10 s before fishing for 2 min. They could not catch any fish within this time as to not influence their behavior by success. To still finish the task with a sense of accomplishment, a fish was placed in the hand-net, when it was underwater for 0.5 s after the 2 min, regardless of the participant’s position.

The difference between the minimum distance to the spider and the minimum distance to the turtle during the 2 min was used as readout of this task.

#### Behavioral Forced-Choice Task (“Path-Choice”)

The participants were standing in a lobby surrounded by a counter and had to move a book from the counter to a shelf (see Figure 1D and Supplementary Video 2). They started by the shelf, facing the counter. In front of them was a table, which could be passed either on the left or on the right side. The spider and the turtle were each placed on one side on the outside table of the counter, so the participants had to walk between one of the animals and the table to get to the book. There were five trials with one book each and the positions of the animals swapped after the first and the third trial. The initial positions were counterbalanced between participants. However, only the book of the first trial was placed in the center. The book was placed to the right in the second trial and to the left in the third trial, and far to the right in the fourth trial and far to the left in the fifth trial (see Figure 1D). In this way, we could add a certain “cost of avoidance” by making one of the paths the shorter or the longer detour compared to the other.

The avoidance score was calculated to quantify the avoidance of the spider considering the cost of avoidance. The sum of the avoided paths was calculated, where the paths were exponentially weighted by the cost of avoidance: 0, if the shorter path was taken; 1, for equal paths; 2, if the shorter detour was taken; 4, if the longer detour was chosen. If the turtle path was chosen, the weight was set negative. Note that for each trial, we evaluated the outward and return journey separately, yielding a sum of ten paths and a score within the range of −14, if they never avoided the spider, and 14, if they always avoided the spider. In this way, the score is symmetrically distributed and indicates the preference for spider or turtle.

#### Behavioral Approach Task (“Touch the Enemy”)

The participants were in a room with a door and a large window with closed blinds (see Figure 1E and Supplementary Video 3). They started at the edge of one wall facing the center of the room. In front of the opposite wall was a small round table at a height of 1.1 m presenting one of the animals at a time. The participants were instructed to walk to the object and touch it with the right hand, as soon as the countdown of 10 s finished. Each animal was presented twice in an alternating manner, resulting in four trials. The animal type of the first trial was counterbalanced between participants.

For each trial, we defined the time to touch as the time from the end of the countdown to the touch of the animal. As readout of this task, we used the difference of the time to touch between the first spider trial and the first turtle trial. A positive readout means that the participant took more time to touch the spider the first time compared to the turtle the first time. Six values in the phobics (21, 22, 30, 39, 70, and 212 s) were truncated to 20 s.

### Statistics

All statistics were performed in MathWorks Matlab R2021a. The Matlab-toolbox “Measures of Effect Size” version 1.6.1 (Hentschke and Stüttgen, 2011) was used to calculate the effect sizes Cohen’s U3 for Mann–Whitney *U*-tests, the η^2^ for analyses of variance, Hedges’ g_1_ for one sample *t*-tests, and Glass’ Δ for two sample *t*-tests. The partial-eta-squared (η${}_{p}^{2}$) and generalized-eta-squared (η^2^_*G*_) were calculated by us for repeated measure analyses of variance (rmANOVA) (Olejnik and Algina, 2003; Bakeman, 2005). In all tests, an alpha level of 0.05 was used for significance. The figures were generated with the Matlab toolbox “Gramm” (Morel, 2018).

#### Physiology

Eye data were recorded in Unity at a sampling rate of approximately 110 Hz using the VIVE Eye and Facial Tracking SDK (SRanipal version 1.3.2.0).

Pupil size was preprocessed in Matlab: first, outliers were removed, defined as values that were more than three scaled absolute deviations away from the moving median with a window size of 100 samples. Then, missing values resulting from outlier detection and closed eyes were linearly interpolated and the pupil size was resampled to regular 110 Hz using the “nearest” method. Finally, the data were rescaled to an interval of zero to one.

The gaze data was recorded in Unity: In each frame, the most recently available eye data from both eyes was used and combined with head position and rotation to determine the direction of gaze in three-dimensional VR. This was used for collision detection with virtual objects to determine the focused object. Later in Matlab, when the time series of the focused object was used to determine the viewing duration and the number of glances at an object of interest, viewing gaps of less than 200 ms, during which no focus on the object was detected, were considered continuous.

The electrocardiography signal was analyzed in Matlab using the PhysioNet-Cardiovascular-Signal-Toolbox (version 1.0.2; Vest et al., 2019) as described in Binder and Spoormaker (2020) resulting in a timeseries of RR intervals, which represent the duration between successive heartbeats, stored at 250 Hz.

#### Behavioral Tasks

Based on the construction, we assumed a continuous scale level for the readouts of the Fishing and Touch the Enemy tasks and an ordinal scale level for the readout of the Path-Choice task. Accordingly, we calculated ANOVAs with *post hoc t*-tests and Pearson correlations for the Fishing and Touch the Enemy tasks. For the Path-Choice tasks we performed the Kruskal–Wallis test with *post hoc* Mann–Whitney-*U* tests and spearman correlations.

In the Fishing task, a repeated measure ANOVA (rmANOVA) was performed with group as the between-factor and stimulus as the within-factor to analyze the number of glances at the stimuli in an explorative manner. *Post hoc*, the within-subject difference between the number of glances at the spider and the number of glances at the turtle was compared between groups using independent *t*-tests and Pearson correlated with the FSQ score. To explore participants orientation during the Fishing task, the mean angle between the hip and the respective stimulus was calculated for each side. An angle of zero or 180 means that the hips were aligned with the front or back side to the stimulus, respectively. As with the analyses of the number of glances, they were analyzed by rmANOVA, independent *t*-tests and Pearson correlation. Four participants (two phobics and two fearfuls) were excluded from these analyses because they never entered the side with the spider. For each participant, the RR interval and pupil size per stimulus side were defined as the mean over time while on the pelvic half with the corresponding stimulus.

To examine Touch the Enemy times in detail, the path from the start position to the stimulus was divided into three equal areas and the time spent in each area was determined. These durations were analyzed by rmANOVA with group as between-factor and trial, stimulus, and area as within-factors. Furthermore, to gain insight into the directness and automation of the approach movements, the changes in acceleration of the right hand were analyzed: First, the irregular right-hand position for each frame were down sampled to regular 10 Hz samples using spline interpolation. Second, the number of sign changes of the second derivative of these data was determined for each trial and used as the number of changes in acceleration. Seven values, in the phobic group (111, 124, 147, 181, 241, 251, and 739) were truncated to 100. The number of changes in acceleration were analyzed with a rmANOVA with group as the between-factor, and trial and stimulus as the within-factor. The pre-touch pupil size was defined as the mean preprocessed pupil size of the right eye in the 0.5 s before the stimulus was touched and analyzed with a rmANOVA with group as the between-factor, and trial and stimulus as the within-factor. One participant of the phobic group was excluded from this analysis as she closed her eyes before touching the spider. Similarly, the pre-touch RR interval was defined as the mean RR interval in the last second before the stimulus was touched and analyzed with a rmANOVA with the same factors. Here, eight participants were excluded because of missing data.

#### Across Tasks Analyses

To investigate the consistency in behavior, Spearman correlations between the readouts of the three behavioral tasks were calculated. Spearman correlations were also used to examine the relations between the behavioral readouts and subjective data from the online home questionnaire and the post-VR questionnaire. To account for multiple testing, the significance level ^****^, representing the conservative Bonferroni corrected *p* < 0.00031 = 0.05/(3 × 54), was added. In addition, as many of the questionnaire scales were also Spearman correlated with the FSQ and the valence rating of the spider, partial Spearman correlations were calculated between the behavioral readouts and the subjective data, controlling for either the FSQ or the valence rating.

## Results

### Manipulation Check

The participants felt highly present in the VR as rated in the Presence Questionnaire 3 (Mean ± SD, range: 1–7, 7 = best): involvement, 5.4 ± 0.7; sensory fidelity, 5 ± 0.8; adaptation immersion, 5.9 ± 0.6; interface quality (1 = best), 2.1 ± 0.9; and in the iGroup Presence Questionnaire (range: 0–6, 6 = best): general presence, 4.5 ± 1.2; spatial presence, 4.8 ± 0.7; involvement, 4.6 ± 1.0; experienced realism, 2.9 ± 1.1. Moreover, they reported only slight side effects in the SSQ (approximate theoretical range: 0–200, 0 = best): total score, 19.7 ± 25.0; nausea, 18.5 ± 30.5; oculomotor symptoms, 12.5 ± 13.5; disorientation, 23.3 ± 34.5.

The difference in the valence ratings of the turtle and the spider was analyzed with the Kruskal–Wallis test and revealed an effect of group [X *^2^*(2, *N* = 31) = 17.2, *p* < 0.001]. The *post hoc* group comparisons revealed significant differences between phobics and fearfuls [*U*(n_1_ = 15, n_2_ = 6) = 76.5, *p* < 0.05, *U3* = 0.07], and between phobics and non-fearfuls [*U*(n_1_ = 15, n_2_ = 10) = 142.5, *p* < 0.001, *U3* = 0.00], but not between fearfuls and non-fearfuls [*U*(n_1_ = 10, n_2_ = 6) = 46, *p* = 0.08, *U3* = 0.08]. The differences in the valence ratings of the stimuli were strongly correlated with the FSQ scores (*r*_*s*_ = 0.67, *p* < 0.001). The relationship is depicted in Figure 2B.

### Behavioral Tasks

#### Fishing

**FIGURE 3:**
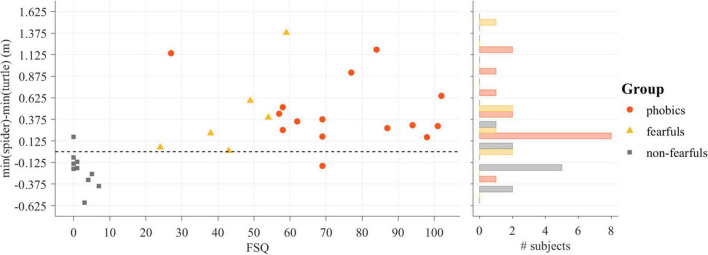
The result of the Fishing task dependent on the Fear of Spiders Questionnaire (FSQ) score (left) and accumulated in a histogram (right).

The individual difference in the minimum distance to the spider and the minimum distance to the turtle during the 120 s was analyzed with an ANOVA. It revealed an effect of group [*F*(2,28) = 11.62, *p* < 0.001, η^2^ = 0.45]. The *post hoc t*-test revealed increased avoidance behavior for phobics compared to non-fearfuls [*t*(23) = 5.17, *p* < 0.001, Δ = 1.74] and for fearfuls compared to non-fearfuls [*t*(14) = 3.65 *p* < 0.01, Δ = 1.21], but no differences between phobics and fearfuls [*t*(19) = 0.09, *p* = 0.93, Δ = 0.05]. The readout of the Fishing task was strongly correlated with the FSQ score (*r* = 0.57, *p* < 0.001). The differences in the minimal distance to the stimuli as related to participants’ FSQ scores and group membership is depicted in Figure 3.

**FIGURE 4:**
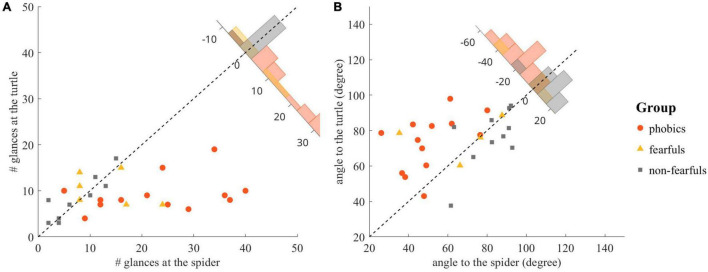
Detailed analyses of the Fishing task: **(A)** Comparison of the number of glances at the spider and the number of glances at the turtle, including the histogram of individual differences in the upper right corner. **(B)** Comparison of the angle between the chest front and the spider and the angle between the chest front and the turtle, including the histogram of individual differences in the upper right corner. The angle was 0 if the chest front was facing the animal and 180 when it was facing away from the animal.

The number of glances at the stimuli was analyzed with an ANOVA and revealed a group effect [*F*(2,28) = 5.82, *p* < 0.01, η${}_{p}^{2}$ = 0.29, η${}_{G}^{2}$ = 0.21], a stimulus effect [*F*(1,28) = 10.87, *p* < 0.01, η${}_{p}^{2}$ = 0.19, η${}_{G}^{2}$ = 0.13], and a stimulus × group interaction [*F*(2,28) = 9.51, *p* < 0.001, η${}_{p}^{2}$ = 0.29, η${}_{G}^{2}$ = 0.20]. The *post hoc* group comparisons revealed an increased number of glances at the spider for phobics compared to fearfuls [*t*(19) = 2.18, *p* < 0.05, Δ = 0.96] and for phobics compared to non-fearfuls [*t*(23) = 4.16 *p* < 0.001, Δ = 1.30], but no differences between fearfuls and non-fearfuls [*t*(14) = 1.36, *p* = 0.19, Δ = 0.42]. The difference in the number of glances at the stimuli was strongly correlated with the FSQ score (*r* = 0.57, *p* < 0.001). The number of glances at the stimuli is depicted in Figure 4A.

The analysis of participants’ orientation toward the stimuli revealed a group effect [*F*(2,24) = 4.48, *p* < 0.05, η${}_{p}^{2}$ = 0.27, η${}_{G}^{2}$ = 0.22], a stimulus effect [*F*(1,24) = 6.13, *p* < 0.05, η${}_{p}^{2}$ = 0.08, η${}_{G}^{2}$ = 0.06], and a stimulus × group interaction [*F*(2,24) = 8.77, *p* < 0.01, η${}_{p}^{2}$ = 0.21, η${}_{G}^{2}$ = 0.16]. The *post hoc* group comparisons revealed a smaller angle toward the spider for phobics compared to non-fearfuls [*t*(21) = −4.53 *p* < 0.001, Δ = −1.69], but no differences between phobics compared to fearfuls [*t*(15) = −1.27, *p* = 0.22, Δ = −0.76] and fearfuls compared to non-fearfuls [*t*(12) = −1.63, *p* = 0.13, Δ = −0.63]. The difference in orientation was strongly correlated with the FSQ score (*r* = −0.63, *p* < 0.001). The orientations are depicted in Figure 4B.

**FIGURE 5:**
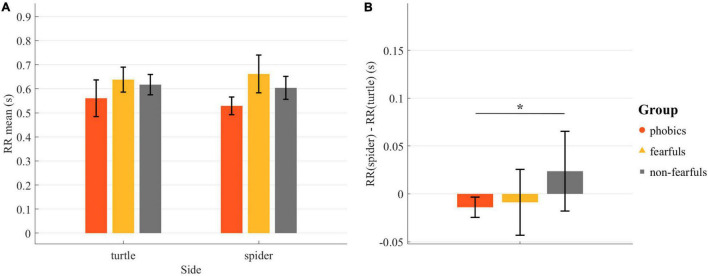
Heart rate analyses during the Fishing task: **(A)** The mean RR interval in seconds per stimulus side while they stayed there. **(B)** The individual differences between the mean RR interval on the spider side and the mean RR interval on the turtle side. Bars = group mean; lines = standard error; RR interval = heartbeat to heartbeat duration; *p < 0.05.

Participants’ heart rate expressed as RR interval per stimulus side during the Fishing task was analyzed with a rmANOVA, which revealed neither a group effect [*F*(2,24) = 3.12, *p* = 0.06, η${}_{p}^{2}$ = 0.21, η${}_{G}^{2}$ = 0.20], nor a stimulus effect [*F*(2,24) = 0.001, *p* = 0.97, η${}_{p}^{2}$ < 0.001, η${}_{G}^{2}$ < 0.001], nor a group × stimulus interaction [*F*(2,24) = 2.85, *p* = 0.08, η${}_{p}^{2}$ = 0.01, η${}_{G}^{2}$ = 0.01]. The heart rate expressed as RR interval is depicted in Figure 5A. Albeit the non-significant group effect, we performed direct group comparisons to explore the trends in the data, well-knowing that these were no regular *post hoc* tests. Figure 5B depicts the spider-turtle differences by group and independent *t*-tests revealed a difference between phobics and non-fearfuls [*t*(21) = −2.22, *p* < 0.05, Δ = −2.07], meaning that RR intervals decreased and thus heart rate was increased in the phobics group when on the side with the spider. We did not find any differences or trends in the mean pupil size (all *p* > 0.50 in rmANOVA).

#### Path-Choice

**FIGURE 6:**
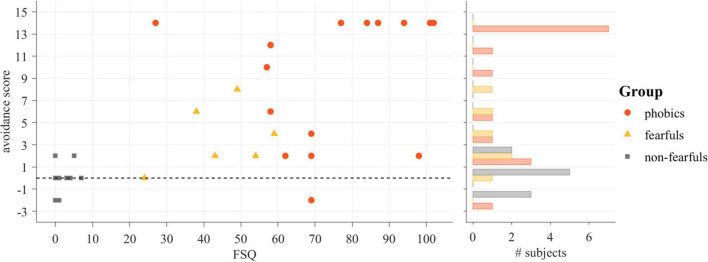
The result of the Path-Choice task dependent on the Fear of Spiders Questionnaire (FSQ) score (left) and accumulated in a histogram (right).

The avoidance score was analyzed with the Kruskal–Wallis test and revealed an effect of group [X *^2^*(2, *N* = 31) = 15.70, *p* < 0.001]. The *post hoc* group comparisons revealed significant differences between phobics and non-fearfuls [*U*(n_1_ = 15, n_2_ = 10) = 138.5, *p* < 0.001, *U3* = 0.07], between fearfuls and non-fearfuls [*U*(n_1_ = 10, n_2_ = 6) = 53.5, *p* < 0.05, *U3* = 0.07], but not between phobics and fearfuls [*U*(n_1_ = 15, n_2_ = 6) = 68, *p* = 0.07, *U3* = 0.27]. The avoidance score was strongly correlated with the FSQ score (*r*_*s*_ = 0.70, *p* < 0.001). The avoidance scores as related to participants’ FSQ scores, and group membership is depicted in Figure 6.

#### Touch the Enemy

**FIGURE 7:**
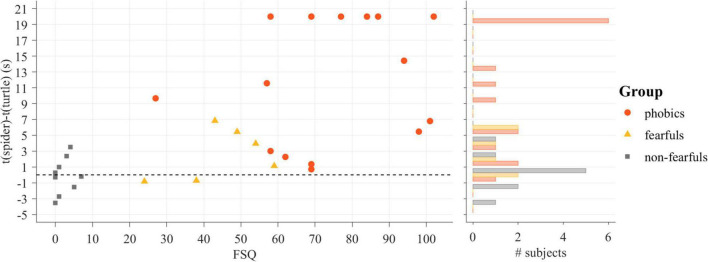
The result of the Touch the Enemy task dependent on the Fear of Spiders Questionnaire (FSQ) score (left) and accumulated in a histogram (right). Individual values were truncated to 20 s.

The difference in the time to touch the spider and the time to touch the turtle was analyzed with an ANOVA and revealed an effect of group [*F*(2,28) = 13.34, *p* < 0.001, η^2^ = 0.49]. The *post hoc t*-tests revealed increased avoidance behavior for phobics compared to fearfuls [*t*(19) = 2.67, *p* < 0.05, Δ = 1.09] and for phobics compared to non-fearfuls [*t*(23) = 4.57 *p* < 0.001, Δ = 1.44], but no differences between fearfuls and non-fearfuls [*t*(14) = 2.07, *p* = 0.06, Δ = 0.81]. The readout of the Touch the Enemy task was strongly correlated with the FSQ score (*r* = 0.67, *p* < 0.001). The differences in the time to touch the stimuli as related to participants’ FSQ scores and group membership is depicted in Figure 7.

**FIGURE 8:**
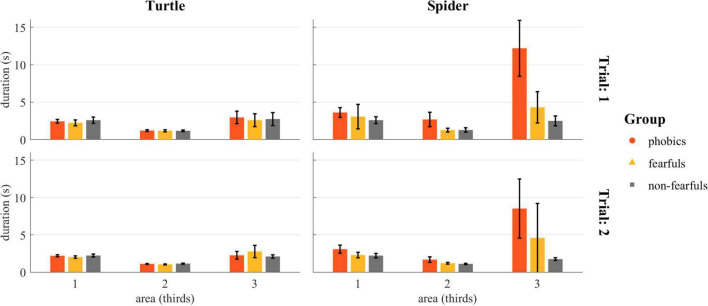
Detailed analyses of the touch duration in the Touch the Enemy task: The duration in seconds the participants stayed on the way to the stimulus in each third. Individual values were truncated to 20 s. The rmANOVA revealed significant main effects of group, trial, stimulus, and area, and significant group × trial, group × stimulus, group × area, group × trial × area, stimulus × area, and group × stimulus × area interactions. Bars = group mean; lines = standard error.

The duration spent in each third during the Touch the Enemy task was analyzed with a rmANOVA and revealed an effect of group [*F*(2,28) = 8.43, *p* < 0.01, η${}_{p}^{2}$ = 0.38, η${}_{G}^{2}$ = 0.12], trial [*F*(2,28) = 11.02, *p* < 0.01, η${}_{p}^{2}$ = 0.05, η${}_{G}^{2}$ = 0.01], stimulus [*F*(2,28) = 12.50, *p* < 0.01, η${}_{p}^{2}$ = 0.27, η${}_{G}^{2}$ = 0.08], and area [*F*(2,56) = 33.63, *p* < 0.001, η${}_{p}^{2}$ = 0.52, η${}_{G}^{2}$ = 0.20]. The interactions with the three largest effects revealed by the rmANOVA were group × area [*F*(4,56) = 9.52, *p* < 0.001, η${}_{p}^{2}$ = 0.38, η${}_{G}^{2}$ = 0.13], group × stimulus [*F*(2,28) = 9.61, *p* < 0.001, η${}_{p}^{2}$ = 0.36, η${}_{G}^{2}$ = 0.12], and group × stimulus × area [*F*(4,56) = 10.56, *p* < 0.001, η${}_{p}^{2}$ = 0.36, η${}_{G}^{2}$ = 0.12]. A complete list of statistics can be found in Supplementary Table 4. The durations are depicted in Figure 8.

**FIGURE 9:**
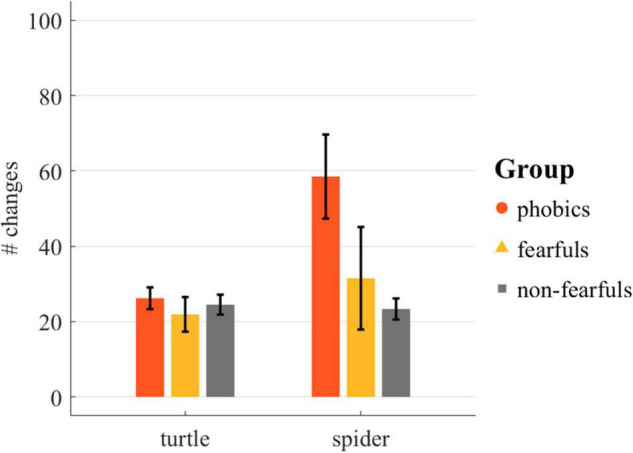
The number of changes in acceleration of the right hand on the way to the stimulus in the Touch the Enemy task. The individual values were truncated to 100. The rmANOVA revealed a significant main effect of group, stimulus, and trial, and a significant group × stimulus interaction. Bars = group mean; lines = standard error.

The number of changes in acceleration was analyzed with a rmANOVA and revealed an effect of group [*F*(2,28) = 8.20, *p* < 0.01, η${}_{p}^{2}$ = 0.37, η${}_{G}^{2}$ = 0.23], trial [*F*(1,28) = 10.06, *p* < 0.01, η${}_{p}^{2}$ = 0.05, η${}_{G}^{2}$ = 0.02], and stimulus [*F*(1,28) = 12.18, *p* < 0.01, η${}_{p}^{2}$ = 0.24, η${}_{G}^{2}$ = 0.13] and a group × stimulus interaction [*F*(2,28) = 8.73, *p* < 0.01, η${}_{p}^{2}$ = 0.31, η${}_{G}^{2}$ = 0.18]. No interactions of group × trial [*F*(2,28) = 1.73, *p* = 0.20, η${}_{p}^{2}$ = 0.02, η${}_{G}^{2}$ = 0.01], stimulus × trial [*F*(1,28) = 0.93, *p* = 0.34, η${}_{p}^{2}$ = 0.01, η${}_{G}^{2}$ < 0.01], or group × stimulus × trial [*F*(2,28) = 0.84, *p* = 0.44, η${}_{p}^{2}$ = 0.01, η${}_{G}^{2}$ = 0.01] were observed. The number of changes in acceleration by stimulus and group is depicted in Figure 9.

**FIGURE 10:**
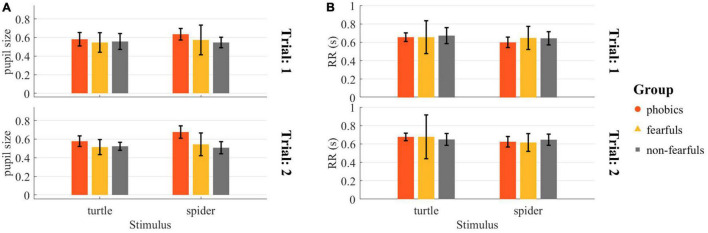
Physiological readouts during the Touch the Enemy task: **(A)** Mean rescaled pupil size during the last 0.5 s before the touch of the stimulus. The rmANOVA revealed a significant group × stimulus interaction. **(B)** Mean RR interval during the last second before the touch of the stimulus. The rmANOVA revealed no significant effects or interactions (all *p* > 0.05). RR interval = heartbeat to heartbeat duration.

The pre-touch pupil size was analyzed with a rmANOVA and revealed a stimulus × group interaction [*F*(2,27) = 3.51, *p* < 0.05, η${}_{p}^{2}$ = 0.05, η${}_{G}^{2}$ = 0.03], showing a stronger pupil dilation to the spider in the phobics group. No other effects or interactions were significant. The pre-touch pupil size by group, stimulus, and trial is depicted in Figure 10A. The pre-touch heart rate expressed as RR interval was also analyzed with a rmANOVA and revealed no significant effects or interactions. However, we also found a trend in the group × stimulus interaction [*F*(2,20) = 1.21, *p* = 0.32, η${}_{p}^{2}$ = 0.02, η${}_{G}^{2}$ = 0.02], indicating a decreased RR interval and thus an increased heart rate to the spider in the phobics group. The pre-touch RR interval by group, stimulus, and trial is depicted in Figure 10B.

### Across Tasks Analyses

**FIGURE 11:**
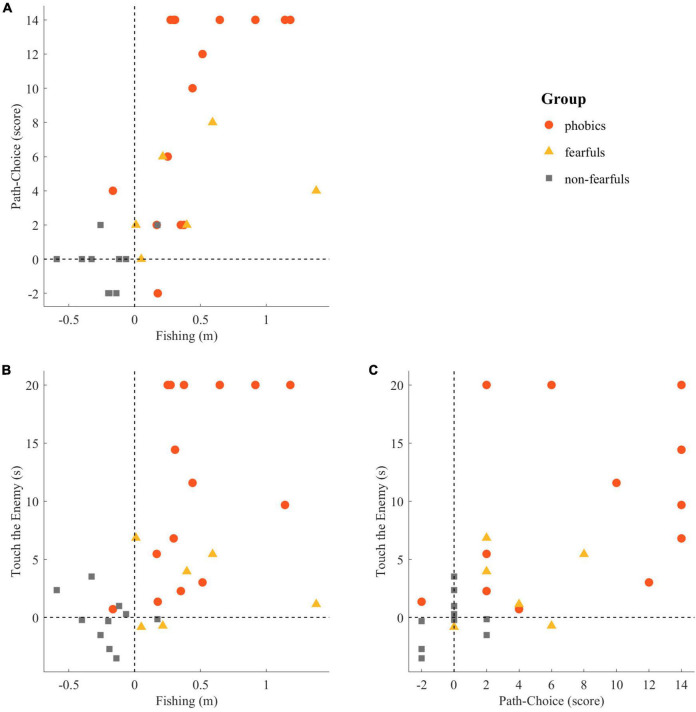
Consistency of behavioral tasks: **(A)** Fishing vs. Path-Choice, **(B)** Fishing vs. Touch the Enemy, and **(C)** Path-Choice vs. Touch the Enemy. Individual values in the Touch the Enemy task were truncated to 20 s. Points represent individual readouts, and dashed lines indicate the boundary between avoidance and approach behavior in each task.

The tasks Fishing and Path-Choice (*r*_*s*_ = 0.75, *p* < 0.001), Fishing and Touch the Enemy (*r*_*s*_ = 0.63, *p* < 0.001), and Path-Choice and Touch the Enemy (*r*_*s*_ = 0.72, *p* < 0.001) were strongly correlated. The tasks consistency is depicted in Figure 11.

The correlations of the behavioral tasks and the questionnaire scales are shown in Table 1. In the last two columns, we added the correlation between the questionnaire scales and either the FSQ or the valence rating of the spider. The visual anxiety scale, the BDI, the valence rating of the spider and some scales of the SSQ and ASI were correlated with the readouts of all behavioral tasks. As these scales were also correlated with the FSQ, partial correlations between the questionnaire scales and the behavioral readouts controlled for the FSQ were calculated. The significant correlations are marked in Table 1 and the details are reported in Supplementary Table 5. Interestingly, when controlling for the valence of the spider, only the two scales nausea (*r*_*s*_ = 0.39, *p* < 0.05) from the SSQ and conscientiousness (*r*_*s*_ = 0.37, *p* < 0.05) from the BFI were significantly partially correlated with the avoidance score of the Path-Choice task, but none of the correlations with the two other tasks survived. A complete list of these partial correlations can be found in Supplementary Table 6.

**TABLE 1:** Spearman correlations between questionnaires and behavioral tasks.

Questionnaire/Scale		Fishing	Path-Choice	Touch the Enemy	FSQ	Valence spider
	Visual anxiety scale	**0.59** ***	0.58***	**0.67** ****	0.53**	−0.67****
	Nervousness	0.30	**0.36** *	**0.32**	0.03	–0.21
	BDI	0.51**	0.31	0.52**	0.56***	−0.57***
	TAI	0.17	–0.03	0.06	0.22	–0.04
	CID-S	0.39*	0.27	0.34	0.54**	–0.32
	SAS	0.67****	0.72****	0.69****	0.93****	−0.75****
	RSES	0.01	**0.07**	–0.11	–0.28	−**0.00**
Valence	Turtle	–0.13	–0.24	0.01	–0.21	0.14
	Spider	−0.75****	−0.81****	−0.80****	−0.78****	**1.00** ****
	Turtle–Spider	**0.70** ****	**0.68** ****	**0.67** ****	0.67****	−0.87****
SSQ	Nausea	**0.59** ***	**0.67** ****	**0.61** ****	0.50**	−0.59***
	Oculomotor	0.32	0.47**	0.52**	0.47**	−0.52**
	Disorientation	0.53**	0.46**	0.48**	0.53**	−0.60***
	Total	0.58***	**0.66** ****	0.65****	0.65****	−0.71****
PQ3	Involvement	0.03	0.06	0.30	0.21	–0.14
	Sensory Fidelity	–0.13	0.00	0.17	0.02	–0.06
	Adaptation Immersion	–0.16	–0.19	–0.03	–0.11	0.09
	Interface Quality	0.10	–0.10	–0.07	–0.05	0.11
IPQ	General presence	–0.06	−**0.01**	0.14	0.33	–0.18
	Spatial presence	–0.21	–0.11	–0.15	–0.17	0.27
	Involvement	0.01	0.06	0.05	0.06	–0.15
	Experienced realism	0.12	0.04	0.12	0.24	–0.15
BFI	Extraversion	0.24	0.25	0.44*	0.28	–0.35
	Agreeableness	–0.10	0.05	0.01	0.04	0.09
	Conscientiousness	0.16	0.26	0.19	0.05	–0.05
	Neuroticism	0.04	–0.00	0.07	0.00	–0.06
	Openness	0.20	0.07	0.29	0.17	–0.26
ASI	Somatic concerns	0.52**	0.47**	0.45*	0.67****	−0.55**
	Social concerns	0.21	0.27	0.48**	0.54**	−0.46**
	Cognitive concerns	0.32	0.16	0.42*	0.45*	–0.30
	Total	0.45*	0.39*	0.52**	0.63****	−0.53**
SSSV	Thrill and Adventure	–0.29	–0.35	–0.18	–0.29	0.23
	Disinhibition	0.17	0.24	0.17	0.50**	–0.30
	Experience Seeking	0.05	0.06	–0.04	–0.13	–0.05
	Boredom Susceptibility	0.03	0.06	0.03	0.31	–0.24
	Total	–0.02	–0.03	–0.02	0.14	–0.12
FEE	Death	0.16	0.26	0.16	0.23	–0.27
	Body Secretions	0.02	−**0.00**	0.35	0.40*	–0.25
	Spoilage	0.15	0.09	0.02	0.30	–0.10
	Hygiene	0.17	0.08	0.16	0.40*	–0.32
	Oral rejection	0.28	0.30	0.22	0.33	−0.41*
	Total	0.18	0.14	0.17	0.37*	–0.30
FKK	Self-concept (SC)	–0.19	–0.06	–0.18	−0.38*	0.20
	Internality (I)	–0.09	0.05	0.03	–0.09	–0.05
	Powerful others (P)	0.19	0.08	0.08	0.34	–0.11
	Chance-control (C)	0.19	0.00	0.03	0.26	–0.08
	SC + I	–0.24	–0.06	–0.18	–0.34	0.16
	P + C	0.20	0.05	0.06	0.34	–0.10
	Total	–0.03	–0.08	0.09	–0.01	0.08
FSQ	Avoidance Coping	0.59***	0.68****	0.67****	0.97****	−0.77****
	Fear of Harm	0.63****	0.71****	0.73****	0.98****	−0.77****
	Total	0.60***	0.70****	0.72****	1.00****	−0.78****
IE4	Internal	0.00	0.12	**0.18**	–0.14	0.01
	External	0.16	0.24	**0.10**	0.45*	–0.31

*p < 0.05; **p < 0.01; ***p < 0.001; ****p < 0.00031. Bold values indicate that the respective partial correlation was significant when controlling for Fear of Spider Questionnaire (FSQ). BDI, Beck-Depression Inventory II; TAI, Trait Anxiety; CID-S, Composite International Diagnostic Screener; SAS, Fear of Spiders Screening; RSES, Rosenberg’s global Self-Esteem; SSQ, Simulator Sickness Questionnaire; PQ3, Presence Questionnaire 3; IPQ, iGroup Presence Questionnaire; BFI, Big Five Inventory; ASI, Anxiety Sensitivity Index 3; SSSV, Sensation Seeking Scales; FEE, assessment of disgust sensitivity; FKK, competence and locus of control; FSQ, Fear of Spider Questionnaire; IE4, Short Scale for the Assessment of Locus of Control.

## Discussion

We evaluated which of the tasks and variables would reveal the most robust differences between phobics and matched healthy controls while simultaneously assessing to what extent participants’ behavior depended on their fear levels. As the healthy controls were quite heterogeneous in terms of their Fear of Spiders Questionnaire (FSQ) scores, we subdivided them into “fearfuls” that had elevated FSQ scores, and “non-fearfuls” that had no fear of spiders. We investigated their behavior in the presence of a spider and a neutral animal and found that phobics strongly disliked the spider in iVR, but fearfuls and non-fearfuls rated the spider only slightly more unpleasant than the neutral animal. Across all tasks, spider phobics showed significant avoidance behavior. In the behavioral search task (Fishing), the phobics and fearfuls strongly avoided the spider, but the non-fearfuls did not. In the behavioral forced-choice task (Path-Choice), the phobics also strongly avoided the spider, but the non-fearfuls did not and the fearfuls showed mild avoidance behavior. In the behavioral approach task (Touch the Enemy), the phobics strongly delayed touching the spider, but the fearfuls and non-fearfuls showed no delay.

The objectively quantified avoidance behavior showed strong correlations with the FSQ in all three tasks. Furthermore, the valence of the spider was strongly correlated with the FSQ and the behavioral readouts of all tasks. This raised the question of the directionality of the effects and prompted us to calculate additional partial correlations. Controlling for the valence rating completely removed the correlation between the FSQ and the behavioral readouts but vice versa the correlations between valence rating and behavioral readouts survived controlling for the FSQ. This suggest that the valence rating of the spider is mediating the relationship between FSQ and avoidance behavior. In addition, the valence of the spider was also strongly correlated with the Visual Anxiety Scale and SSQ, which assessed participants’ subjective well-being after the VR session. This suggests that the valence of the spider was the main factor influencing both the objective avoidance behavior and the subjective experience of the exposure. The appearance of the spider seems to be the key factor in controlling the intensity of exposure as well as in generalizing fear and extinction learning.

On top of using a two-group design based on the spider phobia diagnosis of the Composite International Diagnostic Interview (CIDI), we additionally split up the non-phobics group into non-fearfuls and fearfuls based on participants’ FSQ scores with cut-offs as used in Rinck and Becker (2007), Rinck et al. (2010). This allowed us to better distinguish the fearfuls from phobics and non-fearfuls and provided further insight into the process of adaptive and maladaptive avoidance behavior: while the phobics and non-fearfuls showed consistent avoidance and non-avoidance behavior, respectively, the fearfuls’ behavior varied between tasks: in the search task, they showed strong avoidance behavior like the phobics did, but in the approach task they showed no avoidance behavior, and in the forced-choice task they showed moderate avoidance behavior in between the fearfuls and phobics. This suggests that participants with a medium level of fear avoid a feared stimulus if it is irrelevant to the task and the degree of freedom is high, but fear does not influence their behavior if the feared stimulus is relevant for the task. This is in line with our previous study (Binder and Spoormaker, 2020), in which healthy participants showed the same pattern of avoidance behavior toward fear conditioned stimuli across three behavioral tasks. It underlines that the behavioral search task with stimuli being less relevant for the task is the most sensitive one to detect avoidance behavior and the behavioral approach task is less sensitive, but better suited to detect differences for high levels of fear.

More detailed behavioral analyses within the tasks showed that in the behavioral search task, phobics looked more frequently at the spider than at the neutral stimulus, but the fearfuls and non-fearfuls did not. This is in line with attentional bias theory, which postulates that highly-anxious individuals tend to direct attention to fear-related stimuli, whereas low-anxious subjects do not (Mathews and MacLeod, 1985; MacLeod et al., 1986; Bar-Haim et al., 2007; Abado et al., 2020). Furthermore, we explored participants’ body orientation and observed that the phobics preferred to have the spider in front of them when they searched at the spider’s side. The non-fearfuls and most of the fearfuls did not show this “defensive” behavior. An additional manipulation in the behavioral forced-choice task was the cost of avoidance, which was added in later trials. Despite such costs of avoidance, almost half of the phobics always avoided the spider, but none of the fearfuls or non-fearfuls did. This shows the specific willingness or habit of phobics to accept personal disadvantages to avoid their fear, similarly to what the fearful participants in Pittig et al. (2014) did, when they generally avoided choices associated with pictures of spiders in a gambling task, even when they were offered advantages. In the behavioral approach task when the phobics approached the spider, although they hesitated at the beginning and walked slower in the middle third, the main hesitation occurred in the last third, indicating that fear levels increased with proximity to the spider. This is consistent with the predatory imminence hypothesis, according to which defensive behavior changes depending on the perceived distance to the threat (Fanselow and Lester, 1988). Interestingly, the phobics touched the spider much faster already in the second trial. The explanation given by participants in an unstructured interview at the end of the study was that they “knew” what the spider was doing after the first trial. This raises the question of what they expected beforehand? According to Arntz et al. (1993), beliefs that the spider is coming toward one or jumping onto one as well as self-related beliefs such as losing control are very frequent in spider phobia. This cognitive aspect of specific phobia was also elaborated by Armfield (2006) in the cognitive vulnerability model, according to which perceived controllability, predictability, and dangerousness of a stimulus contribute to the individual’s fear. He further showed, that in an imaginary task, especially the manipulation of perceived controllability or predictability of the spider influenced the task-related spider fear (Armfield, 2007). In a meta-analytic review, Gallagher et al. (2014) showed that lower perceived control was associated with anxiety disorders and that perceived control was an important predictor for cognitive-behavior therapy outcome. Similarly, Tardif et al. (2019) showed that changes in perceived self-efficacy and beliefs about spiders were related to the reductions in fear of spiders after exposure in VR. Transferring these insights to our situation led us to speculate that the knowledge gained might have changed the participants’ beliefs about spiders, increased the perceived control and thus decreased the avoidance behavior.

Additionally, we investigated the physiological effects of the behavioral approach and search tasks. We found increased pupil size and a trend toward increased heart rate when phobics approached the spider. In the behavioral search task, we found a trend toward increased heart rate when phobics searched in the side of the spider, but no effects on pupil size. In summary, we found physiological activation in response to exposure to the feared stimulus as proposed by emotion processing theories (Lang, 1985; Foa and Kozak, 1986; Barlow, 2002) and already shown *in vivo* (Sartory et al., 1977) as well as in VR (Diemer et al., 2014). However, our effects were rather small, and we could not detect group differences in all tasks. We suspect that this is due to difficulties arising from the free movement of participants: As we were interested in overt behavior, the intensity of participants’ body movements varied greatly, which is known to have a strong effect on heart rate (e.g., Hammond and Froelicher, 1985). Likewise in the search task, participants had frequent head movements in response to the task, resulting in large fluctuations in illuminance that affects pupil size (e.g., Watson and Yellott, 2012). However, if the influence of illuminance could be controlled, pupillometry seems to be more sensitive than heart rate for measuring fear, as suggested by the larger effect sizes of pupillometry compared with heart rate in the behavioral approach task. In this study, we had 98% power to detect large effects and 14% power to detect small effects, so if physiological effects are indeed smaller, we simply need larger studies, although this is not necessary for the behavioral effects.

In contrast to our previous study (Binder and Spoormaker, 2020), in which we did not find any reliable relationship between avoidance behavior and the Beck-Depression Inventory or the scales of the Anxiety Sensitivity Index, we detected now several correlations between these variables. However, these questionnaire scales were also correlated with the FSQ, and when we controlled for this, there were no other correlations. This indicates that these traits had no direct influence on avoidance behavior but were rather related to spider phobia, fitting the positive association of specific phobia with both comorbid depression (Choy et al., 2007a; Lieb et al., 2016) and anxiety sensitivity (Olatunji and Wolitzky-Taylor, 2009). Another explanation could be that in our previous study, we included only healthy participants, which resulted in low variance in the questionnaire scales. By including spider phobic participants in this study, we increased the variance and thus improved the detection of the correlations.

We also aimed to investigate the feasibility of the procedure and its tasks, as confronting a phobic with the feared animal might be critical, especially in a fully automated setup. Although we included phobics who reported severe problems with spiders in the CIDI, all participants were able to complete the procedure without manual intervention. Even in the behavioral approach task, all participants were able to touch the spider. This is surprising considering that even after *in vivo* exposure, 10–20% of patients are unable to do so (Choy et al., 2007b). In the unstructured interview at the end of the procedure, the phobics reported that this was possible because it was not a real spider, which is in line with the moderate rating of the experienced realism in the iGroup Questionnaire. Nevertheless, they did show robust avoidance behavior and strong subjective fear. This is a key element of iVR: Although it is clearly an artificial environment, it triggers real emotions and real behavior. This is reflected in the effects of VR exposure therapy, which are a similar size to the effects of exposure therapy *in vivo* (Carl et al., 2019). Moreover, compared to *in vivo* exposure, iVR has the advantages that it is highly standardized, and the feared stimulus can be flexibly adapted to the patient’s phobia without the need for maintenance and upkeep. Our results further enhance VR exposure therapy by providing multiple objective measures such as distance, choice, timing, eye gaze, body orientation, and hand movements, allowing for a holistic quantification of momentary fear levels that can be determined online and used to automatically adjust the intensity of exposure. In addition, we have demonstrated the feasibility of a fully automated iVR procedure with several degrees of exposure for patients with severe specific phobia. In this way, our findings could contribute to an efficient, fully automated, and accepted therapy for specific phobia that provides not only talk therapy but also training in real-life situations with direct active learning. No costly trained therapists are needed making it easier to scale up and offer therapy to more people (Freeman et al., 2018).

A limitation of this study is the rather small sample size. Therefore, we were only able to detect large effects and we cannot say anything about small or moderate effects. Future research should aim at larger sample sizes, especially when interested in physiological readouts. Another limitation is the restriction to female participants only, which limits the generalization to male subjects.

In summary, phobics successfully managed all tasks and showed consistent avoidance behavior across all behavioral tasks in iVR, which was also reflected in eye gaze, body orientation, and hesitation. Participants entirely without fear of spiders consistently showed no avoidance behavior across all tasks. Non-phobic participants with subthreshold moderate levels of phobic fear showed some avoidance behavior depending on the tasks, which differed in the task relevance of the spider and the degrees of freedom. Additionally, we identified subjective valence ratings of the spider as main influence on both objective avoidance behavior and subjective well-being after exposure, independent of general phobic-fear levels. Patients could benefit from this study in two ways: First, the holistic quantification of the momentary fear level allows for a more precise adjustment of the intensity of the exposure, thus improving the acceptance and efficiency of the therapy. Second, the discussed possible influences of perceived control and appearance of the feared stimulus might provide additional concepts to work on in therapy.

## Data Availability Statement

The original contributions presented in the study are included in the article/Supplementary Material, further inquiries can be directed to the corresponding author/s.

## Ethics Statement

The studies involving human participants were reviewed and approved by the Local Ethics Committee at the Faculty of Medicine at Ludwig Maximilian University of Munich. The patients/participants provided their written informed consent to participate in this study.

## Author Contributions

FB and VS designed the study with support of PZ and DP. FB developed and implemented the tasks, recruited the participants, acquired, and analyzed the data. FB and VS interpreted the data. FB wrote the manuscript under supervision of VS and contribution of PZ and DP. All authors read and approved the submitted version.

## Conflict of Interest

VS has provided consulting and advisory services for Roche. PZ has received speaker fees from Pfizer, Servier, Hexal, neuraxpharm, and Trommsdorff, also part of the advisory board of Pfizer and Servier, and also received funding from Servier. The aforementioned disclosures are unrelated to this study. The remaining authors declare that the research was conducted in the absence of any commercial or financial relationships that could be construed as a potential conflict of interest.

## Publisher’s Note

All claims expressed in this article are solely those of the authors and do not necessarily represent those of their affiliated organizations, or those of the publisher, the editors and the reviewers. Any product that may be evaluated in this article, or claim that may be made by its manufacturer, is not guaranteed or endorsed by the publisher.

## Supplementary Material

Supplementary Data Sheet 1 Supplementary tables and figures.

Supplementary Data Sheet 2 Underlying data.

Supplementary Video 1 Video of the Fishing task.

Supplementary Video 2 Video of the Path-Choice task.

Supplementary Video 3 Video of the Touch the Enemy task.
