# Supplementary material for: Facing Your Fear in Immersive Virtual Reality: Avoidance Behavior in Specific Phobia
Source: Front Behav Neurosci. 2022 Apr 27;16:827673. doi: 10.3389/fnbeh.2022.827673 (PMC9094686; doi:10.3389/fnbeh.2022.827673)
Supplement: Supplementary Data Sheet 1 — Supplementary tables and figures. [file Data_Sheet_1.PDF]

Supplementary Material

1 Comorbidities

According to the Composite International Diagnostic Interview (CIDI), all participants in the phobics group fulfilled the DSM-IV criteria for animal type phobia for spiders. Five of them fulfilled the criteria for one or two comorbid anxiety disorders (mostly other types of phobias, once agoraphobia and once panic-attacks). The slightly increased BDI and Composite International Diagnostic Screener (CID-S) scores in this group (see Supplementary Table 1) might indicate that there are a few participants who might have further mental comorbidities. All participants in the non-anxiety group were most likely free of mental disorders, as no diagnoses were made in the CIDI and BDI and CID-S scores were very low. See also Supplementary Table 2 for detailed numbers of diagnoses resulting from the CIDI (anxiety section only).

2 Supplementary Data

Supplementary Table 1. Overview of demographic data and questionnaires for each group. *M* = mean, *SD* = standard deviation
BDI: Beck-Depression Inventory II, TAI: Trait Anxiety, CID-S: Composite International Diagnostic Screener, SAS: Fear of Spiders Screening, RSES: Rosenberg's global Self-Esteem, SSQ: Simulator Sickness Questionnaire, PQ3: Presence Questionnaire 3, IPQ: iGroup Presence Questionnaire, BFI: Big Five Inventory, ASI: Anxiety Sensitivity Index 3, SSSV: Sensation Seeking Scales, FEE: assessment of disgust sensitivity, FKK: competence and locus of control, FSQ: Fear of Spider Questionnaire, IE4: Short Scale for the Assessment of Locus of Control

**Questionnaire / Scale**		**phobics**		**fearfuls**		**non-fearfuls**	
		15		*6*		*10*	
		M	***SD***	***M***	***SD***	***M***	***SD***
	Age	24.93	4.96	24.00	5.40	24.30	2.54
	Body Size	1.68	0.04	1.72	0.04	1.70	0.08
	Weight	60.00	8.67	61.50	5.28	67.20	12.57
	Visual Anxiety Scale	3.47	3.18	2.67	1.21	0.40	0.52
	Nervousness	3.53	3.09	3.33	2.34	2.80	2.30
	BDI	8.80	7.36	9.83	16.02	2.40	2.72
	TAI	39.20	9.60	47.17	11.79	36.00	6.93
	CID-S	2.53	2.61	1.67	1.51	0.50	0.53
	SAS	21.87	2.39	16.17	4.40	3.10	2.73
	RSES	32.67	6.80	30.67	6.71	34.40	5.56
Valence	Turtle	1.47	2.07	3.33	1.03	7.90	1.97
	Spider	9.07	1.94	7.17	2.14	9.90	0.32
	Turtle - Spider	7.60	2.61	3.83	2.64	2.00	2.00
SSQ	Nausea	32.44	38.97	7.95	11.15	3.82	4.93
	Oculomotor	18.19	15.11	12.63	12.38	3.79	5.36
	Disorientation	35.26	43.34	25.52	25.54	4.18	6.72
	Total	31.17	30.93	16.21	13.52	4.49	3.44
PQ3	Involvement	5.65	0.57	4.72	0.26	5.39	0.74
	Sensory Fidelity	5.08	0.79	4.47	0.32	5.18	0.86
	Adaptation Immersion	5.98	0.55	5.27	0.41	6.11	0.47
	Interface Quality	2.02	1.09	2.56	0.50	1.93	0.78
IPQ	General presence	4.80	1.32	3.67	1.03	4.40	0.97
	Spatial presence	4.76	0.74	4.47	0.65	5.14	0.57

	Involvement	4.77	0.85	4.33	1.03	4.42	1.11
	Experienced realism	3.22	1.11	2.12	1.01	2.90	0.95
BFI	Extraversion	3.82	0.63	3.29	1.16	3.44	0.55
	Agreeableness	3.71	0.44	3.57	0.62	3.64	0.57
	Conscientiousness	3.52	0.62	2.96	0.96	3.47	0.62
	Neuroticism	3.05	0.95	3.50	0.73	2.99	0.73
	Openness	3.79	0.62	3.83	0.52	3.63	0.57
ASI	Somatic concerns	1.58	0.65	1.61	0.44	0.77	0.45
	Social concerns	1.04	0.64	0.89	0.34	0.60	0.45
	Cognitive concerns	1.24	0.82	0.75	0.29	0.65	0.37
	Total	1.29	0.62	1.08	0.30	0.67	0.37
SSSV	Thrill and Adventure	5.80	2.37	6.83	1.47	7.80	2.20
	Disinhibition	5.73	2.09	5.00	3.16	3.50	2.01
	Experience Seeking	6.20	1.47	7.17	2.32	6.60	2.27
	Boredom Susceptibility	3.40	1.84	4.00	3.03	2.60	1.51
	Total	21.13	5.07	23.00	7.04	20.50	5.93
FEE	Death	2.17	1.21	3.26	1.53	1.49	0.72
	Body Secretions	3.69	0.52	3.38	0.61	3.37	0.45
	Spoilage	3.57	0.53	3.56	0.43	3.34	0.51
	Hygiene	3.46	0.77	3.35	0.71	2.94	0.73
	Oral rejection	3.99	0.87	3.69	0.99	3.22	0.91
	Total	3.37	0.58	3.44	0.76	2.88	0.53
FKK	Self-concept (SC)	3.60	0.73	3.62	0.56	4.17	0.84
	Internality (I)	4.10	0.62	3.81	0.47	4.26	0.49
	Powerful others (P)	3.27	0.66	3.04	0.62	2.77	0.69
	Chance-control (C)	2.98	0.83	3.12	0.57	2.79	0.60
	SC + I	3.85	0.41	3.72	0.48	4.22	0.57
	P + C	3.12	0.72	3.08	0.57	2.78	0.57
	Total	3.49	0.35	3.40	0.15	3.50	0.34
FSQ	Avoidance Coping	38.00	9.43	25.33	7.00	1.10	1.73
	Fear of Harm	36.13	13.13	19.17	6.77	1.00	1.15
	Total	74.13	20.69	44.50	12.53	2.10	2.51
IE4	Internal	4.27	0.59	3.75	1.21	4.45	0.50
	External	2.20	0.73	2.42	0.66	1.80	0.26

Supplementary Table 2. Comorbidities: The number of participants according to the number of anxiety diagnoses resulting from the CIDI.

	**Number of participants**			
	**0 diagnosis**	**1 diagnosis**	**2 diagnoses**	**3 diagnoses**
phobics	0	10	1	4
fearfuls	3	2	1	0
non-fearfuls	10	0	0	0

Supplementary Table 3. Descriptions of the questionnaires used in this study.

Questionnaire			Description	Range
ASI	Anxiety Sensitivity Index 3	(Kemper et al., 2009)	Anxiety sensitivity is the fear of anxiety symptoms due to concerns about their physical, social, or cognitive harmfulness.	0 - 4
BDI	Beck-Depression Inventory II	(Kühner et al., 2007)	Measures the severity of depressive symptoms.	0 - 63
BFI	Big Five Inventory	(Rammstedt and Danner, 2017)	Inventory to assess the Big Five (OCEAN) personality traits.	1 - 5
CID-S	Composite International Diagnostic Screener	(Wittchen et al., 1999)	A 12-item self-report questionnaire based on the core diagnostic questions of the Composite International Diagnostic Interview, a time-efficient diagnostic screening tool for most DSM-IV and ICD-10 mental disorders.	0 - 12
FEE	assessment of disgust sensitivity	(Schienle et al., 2002)	Questionnaire for the assessment of disgust sensitivity.	1 – 5
FKK	competence and locus of control	(Krampen, 1991)	A questionnaire to assess generalized self-concept of own abilities, internality in generalized control beliefs, socially conditioned externality, and fatalistic externality	1 - 6
FSQ	Fear of Spider Questionnaire	(Szymanski and O'Donohue, 1995; Rinck et al., 2002)	An 18-item self-report questionnaire designed to assess spider phobia.	Fear of Harm: 0 - 60<br>Avoidance: 0 - 48<br>Total: 0 - 108
IE-4	The Internal-External Control Belief Scale	(Kovaleva et al., 2014)	A brief scale for measuring locus of control beliefs.	1 - 5
IPQ	iGroup Presence Questionnaire	(Schubert et al., 1999)	A 14-item questionnaire to measure the subjective sense of being in the virtual environment.	0 - 6
PQ3	Presence Questionnaire 3	(Witmer et al., 2005)	A 32-item questionnaire to measure the subjective sense of being in the virtual environment.	1 - 7
RSES	Rosenberg's global Self-Esteem	(Ferring and Filipp, 1996)	A psychological questionnaire designed to assess a person's self-esteem through self-assessment.	10 - 40
SAS	Fear of Spiders Screening	(Rinck et al., 2002)	A four-item questionnaire designed for efficient screening for fear of spiders.	0 - 24

SSQ	Simulator Sickness Questionnaire	(Kennedy et al., 1993)	A 21-item questionnaire to assess severity of cybersickness experienced during the virtual reality session.	Nausea: 0 – 200<br>Oculomotor: 0 – 159<br>Disorientation: 0 – 292<br>Total: 0 – 236
SSSV	Sensation Seeking Scales, Form V	(Beauducel et al., 2003)	A questionnaire with 40 forced-choice questions for sensation seeking to determine individual levels in sensory stimulation preferences.	0 – 10<br>Total: 0 – 40
TAI	Trait Anxiety	(Spielberger, 1983)	Trait anxiety or anxiety level as a personal characteristic.	20 - 80

Supplementary Table 4. Result of the rmANOVA of the duration spent in each third during the **Touch the Enemy** task with group as between-factor and trial, stimulus, and area as within-factors.
η_p^2: partial eta squared; η_G^2: generalized eta squared

	**SumSq**	**DF**	**MeanSq**	**F**	**p**	η_p^2	η_G^2
(Intercept)	2301.48	1	2301.48	167.09	0.001	0.86	0.58
group	232.28	2	116.14	8.43	0.001	0.38	0.12
Error	385.67	28	13.77				
(Intercept):trial	20.46	1	20.46	11.02	0.003	0.05	0.01
group:trial	14.07	2	7.04	3.79	0.03	0.04	0.01
Error(trial)	51.96	28	1.86				
(Intercept):stimulus	143.92	1	143.92	12.50	0.001	0.27	0.08
group:stimulus	221.29	2	110.64	9.61	0.001	0.36	0.12
Error(stimulus)	322.30	28	11.51				
(Intercept):area	417.87	2	208.93	33.63	< 0.001	0.52	0.20
group:area	236.68	4	59.17	9.52	< 0.001	0.38	0.13
Error(area)	347.91	56	6.21				
(Intercept):trial:stimulus	5.80	1	5.80	3.06	0.09	0.01	0.00
group:trial:stimulus	9.63	2	4.82	2.54	0.10	0.02	0.01
Error(trial:stimulus)	53.07	28	1.90				
(Intercept):trial:area	5.73	2	2.86	1.78	0.18	0.01	0.00
group:trial:area	17.13	4	4.28	2.67	0.04	0.04	0.01
Error(trial:area)	89.89	56	1.61				
(Intercept):stimulus:area	123.01	2	61.51	11.94	< 0.001	0.24	0.07
group:stimulus:area	217.51	4	54.38	10.56	< 0.001	0.36	0.12
Error(stimulus:area)	288.49	56	5.15				
(Intercept):trial:stimulus:area	2.17	2	1.09	0.56	0.57	0.01	0.00
group:trial:stimulus:area	8.93	4	2.23	1.16	0.34	0.02	0.01
Error(trial:stimulus:area)	107.82	56	1.93				

Supplementary Table 5. Partial Spearman correlations between questionnaires and behavioral tasks controlled for the **Fear of Spider Questionnaire** total score.
* = p < 0.05; ** = p < 0.01; *** = p < 0.001; **** = p < 0.00031.
BDI: Beck-Depression Inventory II, TAI: Trait Anxiety, CID-S: Composite International Diagnostic Screener, SAS: Fear of Spiders Screening, RSES: Rosenberg's global Self-Esteem, SSQ: Simulator Sickness Questionnaire, PQ3: Presence Questionnaire 3, IPQ: iGroup Presence Questionnaire, BFI: Big Five Inventory, ASI: Anxiety Sensitivity Index 3, SSSV: Sensation Seeking Scales, FEE: assessment of disgust sensitivity, FKK: competence and locus of control, FSQ: Fear of Spider Questionnaire, IE4: Short Scale for the Assessment of Locus of Control

**Questionnaire / Scale**		**Fishing**	**Path-Choice**	**Touch the Enemy**	**Valence Spider**
	Visual Anxiety Scale	0.41 *	0.35	0.49 **	-0.48 **
	Nervousness	0.36	0.48 **	0.44 *	-0.31
	BDI	0.25	-0.14	0.20	-0.25
	TAI	0.04	-0.26	-0.14	0.21
	CID-S	0.09	-0.19	-0.09	0.19
	SAS	0.36	0.28	0.08	-0.12
	RSES	0.23	0.39 *	0.13	-0.37 *
Valence	Turtle	-0.01	-0.14	0.24	-0.03
	Spider	-0.56 **	-0.59 ***	-0.56 **	1.00 ****
	Turtle - Spider	0.50 **	0.39 *	0.37 *	-0.75 ****
SSQ	Nausea	0.42 *	0.51 **	0.42 *	-0.37 *
	Oculomotor	0.06	0.22	0.30	-0.28
	Disorientation	0.31	0.15	0.17	-0.35
	Total	0.31	0.39 *	0.35	-0.42 *
PQ3	Involvement	-0.12	-0.12	0.22	0.03
	Sensory Fidelity	-0.17	-0.01	0.22	-0.07
	Adaptation Immersion	-0.11	-0.15	0.08	-0.01
	Interface Quality	0.16	-0.10	-0.05	0.13
IPQ	General presence	-0.35	-0.36 *	-0.14	0.14
	Spatial presence	-0.14	0.01	-0.04	0.23
	Involvement	-0.03	0.03	0.01	-0.17
	Experienced realism	-0.03	-0.19	-0.09	0.06
BFI	Extraversion	0.10	0.08	0.35	-0.22
	Agreeableness	-0.15	0.04	-0.02	0.18
	Conscientiousness	0.16	0.31	0.21	-0.01
	Neuroticism	0.05	-0.00	0.10	-0.10
	Openness	0.12	-0.08	0.25	-0.20
ASI	Somatic concerns	0.20	0.01	-0.07	-0.06
	Social concerns	-0.17	-0.17	0.16	-0.08
	Cognitive concerns	0.07	-0.24	0.16	0.08
	Total	0.11	-0.10	0.12	-0.07
SSSV	Thrill and Adventure	-0.15	-0.22	0.05	0.01
	Disinhibition	-0.20	-0.18	-0.32	0.18
	Experience Seeking	0.16	0.22	0.08	-0.26
	Boredom Susceptibility	-0.21	-0.23	-0.29	0.00
	Total	-0.13	-0.18	-0.18	-0.01
FEE	Death	0.03	0.14	-0.01	-0.15

	Body Secretions	-0.31	-0.43 *	0.10	0.11
	Spoilage	-0.04	-0.17	-0.29	0.22
	Hygiene	-0.10	-0.31	-0.20	-0.00
	Oral rejection	0.12	0.10	-0.01	-0.27
	Total	-0.05	-0.18	-0.14	-0.02
FKK	Self-concept (SC)	0.05	0.31	0.15	-0.17
	Internality (I)	-0.05	0.15	0.13	-0.19
	Powerful others (P)	-0.02	-0.24	-0.26	0.26
	Chance-control (C)	0.04	-0.26	-0.23	0.20
	SC + I	-0.04	0.27	0.11	-0.18
	P + C	-0.01	-0.29	-0.29	0.28
	Total	-0.03	-0.10	-0.12	0.11
FSQ	Avoidance Coping	0.03	0.05	-0.13	-0.10
	Fear of Harm	0.22	0.16	0.21	-0.06
IE4	Internal	0.11	0.31	0.40 *	-0.17
	External	-0.16	-0.11	-0.36 *	0.08

Supplementary Table 6. Partial Spearman correlations between questionnaires and behavioral tasks controlled for the **valence rating of the spider**.
* = $p < 0.05$; ** = $p < 0.01$; *** = $p < 0.001$; **** = $p < 0.00031$.
BDI: Beck-Depression Inventory II, TAI: Trait Anxiety, CID-S: Composite International Diagnostic Screener, SAS: Fear of Spiders Screening, RSES: Rosenberg's global Self-Esteem, SSQ: Simulator Sickness Questionnaire, PQ3: Presence Questionnaire 3, IPQ: iGroup Presence Questionnaire, BFI: Big Five Inventory, ASI: Anxiety Sensitivity Index 3, SSSV: Sensation Seeking Scales, FEE: assessment of disgust sensitivity, FKK: competence and locus of control, FSQ: Fear of Spider Questionnaire, IE4: Short Scale for the Assessment of Locus of Control

**Questionnaire / Scale**		**Fishing**	**Path-Choice**	**Touch the Enemy**	**FSQ**
	Visual Anxiety Scale	0.19	0.10	0.31	0.02
	Nervousness	0.22	0.33	0.26	-0.23
	BDI	0.15	-0.30	0.14	0.24
	TAI	0.20	-0.11	0.05	0.30
	CID-S	0.23	0.01	0.14	0.49 **
	SAS	0.23	0.29	0.21	0.84 ****
	RSES	0.01	0.12	-0.19	-0.45 *
Valence	Turtle	-0.03	-0.22	0.22	-0.15
	Turtle - Spider	0.14	-0.09	-0.10	-0.03
SSQ	Nausea	0.28	0.39 *	0.28	0.07
	Oculomotor	-0.12	0.09	0.20	0.11
	Disorientation	0.15	-0.04	-0.00	0.13
	Total	0.11	0.22	0.19	0.22
PQ3	Involvement	-0.11	-0.09	0.31	0.15
	Sensory Fidelity	-0.26	-0.07	0.20	-0.04
	Adaptation Immersion	-0.14	-0.20	0.07	-0.08
	Interface Quality	0.28	-0.02	0.04	0.07
IPQ	General presence	-0.30	-0.27	-0.00	0.31
	Spatial presence	-0.01	0.19	0.13	0.08
	Involvement	-0.15	-0.10	-0.13	-0.10
	Experienced realism	0.01	-0.15	-0.01	0.20
BFI	Extraversion	-0.03	-0.05	0.28	0.01
	Agreeableness	-0.05	0.20	0.13	0.17
	Conscientiousness	0.19	0.37 *	0.25	0.02
	Neuroticism	-0.01	-0.09	0.04	-0.08
	Openness	0.02	-0.24	0.15	-0.04
ASI	Somatic concerns	0.20	0.05	0.01	0.46 **
	Social concerns	-0.23	-0.19	0.21	0.33
	Cognitive concerns	0.14	-0.16	0.30	0.35
	Total	0.09	-0.08	0.18	0.42 *
SSSV	Thrill and Adventure	-0.18	-0.29	0.01	-0.18
	Disinhibition	-0.09	-0.00	-0.12	0.46 *
	Experience Seeking	0.01	0.03	-0.14	-0.28
	Boredom Susceptibility	-0.23	-0.24	-0.28	0.20
	Total	-0.16	-0.22	-0.20	0.08
FEE	Death	-0.07	0.07	-0.10	0.03
	Body Secretions	-0.27	-0.36	0.26	0.34
	Spoilage	0.11	0.02	-0.10	0.35
	Hygiene	-0.11	-0.32	-0.17	0.26
	Oral rejection	-0.04	-0.07	-0.20	0.01

	Total	-0.07	-0.19	-0.12	0.22
FKK	Self-concept (SC)	-0.07	0.18	-0.02	-0.36 *
	Internality (I)	-0.19	0.02	-0.01	-0.20
	Powerful others (P)	0.16	-0.03	-0.03	0.41 *
	Chance-control (C)	0.20	-0.10	-0.06	0.32
	SC + I	-0.18	0.12	-0.08	-0.35
	P + C	0.19	-0.06	-0.04	0.42 *
	Total	0.04	-0.02	-0.04	0.08
FSQ	Avoidance Coping	0.03	0.16	0.13	0.92 ****
	Fear of Harm	0.11	0.22	0.29	0.95 ****
	Total	0.04	0.18	0.24	1.00 ****
IE4	Internal	0.01	0.22	0.30	-0.21
	External	-0.12	-0.01	-0.26	0.36

3 References

Beauducel, A., Strobel, A., and Brocke, B. (2003). Psychometrische Eigenschaften und Normen einer deutschsprachigen Fassung der Sensation Seeking-Skalen, Form V. *Diagnostica* 49, 61–72. doi:10.1026//0012-1924.49.2.61.

Ferring, D., and Filipp, S. H. (1996). Messung des selbstwertgefühls: Befunde zu reliabilität, validität und stabilität der Rosenberg-skala. *Diagnostica* 42, 284–292.

Kemper, C. J., Ziegler, M., and Taylor, S. (2009). Überprüfung der psychometrischen Qualität der deutschen Version des Angstsensitivitätsindex-3. *Diagnostica* 55, 223–233. doi:10.1026/0012-1924.55.4.223.

Kennedy, R. S., Lane, N. E., Berbaum, K. S., and Lilienthal, M. G. (1993). Simulator Sickness Questionnaire: An Enhanced Method for Quantifying Simulator Sickness. *Int. J. Aviat. Psychol.* 3, 203–220. doi:10.1207/s15327108ijap0303_3.

Kovaleva, A., Beierlein, C., Kemper, C. J., and Rammstedt, B. (2014). Eine Kurzskala zur Messung von Kontrollüberzeugung: Die Skala Internale-Externale-Kontrollüberzeugung-4 (IE-4). *Zusammenstellung sozialwissenschaftlicher Items und Skalen*. doi:https://doi.org/10.6102/zis184.

Krampen, G. (1991). *FKK - Fragebogen zu Kompetenz- und Kontrollüberzeugungen*. Hogrefe Verlag GmbH & Co. KG.

Kühner, C., Bürger, C., Keller, F., and Hautzinger, M. (2007). Reliabilität und validität des revidierten Beck- Depressionsinventars (BDI-II). Befunde aus deutschsprachigen stichproben. *Nervenarzt* 78, 651–656. doi:10.1007/s00115-006-2098-7.

Rammstedt, B., and Danner, D. (2017). Die Facettenstruktur des Big Five Inventory (BFI). *Diagnostica* 63, 70–84. doi:10.1026/0012-1924/a000161.

Rinck, M., Bundschuh, S., Engler, S., Müller, A., Wissmann, J., Ellwart, T., et al. (2002). Reliabilität und Validität dreier Instrumente zur Messung von Angst vor Spinnen. *Diagnostica* 48, 141–149. doi:10.1026//0012-1924.48.3.141.

Schienle, A., Walter, B., Stark, R., and Vaitl, D. (2002). Ein Fragebogen zur Erfassung der Ekelempfindlichkeit (FEE). *Z. Klin. Psychol. Psychother.* 31, 110–120. doi:10.1026/0084-5345.31.2.110.

Schubert, T., Friedmann, F., and Regenbrecht, H. (1999). “Embodied Presence in Virtual Environments,” in *Visual Representations and Interpretations* (London: Springer London), 269–278. doi:10.1007/978-1-4471-0563-3_30.

Spielberger, C. D. (1983). State-Trait Anxiety Inventory (STAI). *Mind Gard.* 94061, 261–3500. doi:10.1002/9780470479216.corpsy0943.

Szymanski, J., and O’Donohue, W. (1995). Fear of Spiders Questionnaire. *J. Behav. Ther. Exp. Psychiatry* 26, 31–34. doi:10.1016/0005-7916(94)00072-T.

Witmer, B. G., Jerome, C. J., and Singer, M. J. (2005). The Factor Structure of the Presence Questionnaire. *Presence* 14, 298–312.

Wittchen, H.-U., Höfler, M., Gander, F., Pfister, H., Storz, S., Üstün, B., et al. (1999). Screening

for mental disorders: performance of the Composite International Diagnostic – Screener (CID–S). *Int. J. Methods Psychiatr. Res.* 8, 59–70. doi:10.1002/mpr.57.
